# Dual Preconditioning: A Novel Strategy to Withstand Mesenchymal Stem Cells against Harsh Microenvironments

**DOI:** 10.15171/apb.2018.054

**Published:** 2018-08-29

**Authors:** Hamed Bashiri, Fatemeh Amiri, Ali Hosseini, Masoud Hamidi, Amaneh Mohammadi Roushandeh, Yoshikazu Kuwahara, Mohammad Ali Jalili, Mehryar Habibi Roudkenar

**Affiliations:** ^1^Department of Medical Laboratory Sciences, Faculty of Paramedical, Kurdistan University of Medical Sciences, Sanandaj, Iran.; ^2^Department of Medical Laboratory Sciences, School of Paramedicine, Hamadan University of Medical Sciences, Hamadan, Islamic Republic of Iran.; ^3^Medical Biotechnology Research Center, Paramedicine Faculty, Guilan University of Medical Sciences, Rasht, Iran.; ^4^Division of Radiation Biology and Medicine, Faculty of Medicine, Tohoku Medical and Pharmaceutical University, Sendai, Japan.; ^5^Cardiovascular Disease Research Center, Department of Cardiology, Heshmat Hospital, School of Medicine, Guilan University of Medical Sciences, Rasht, Iran.

**Keywords:** Hydrogen peroxide, Mesenchymal Stem Cells, Serum deprivation, Simultaneous preconditioning, Survival, Harsh microenvironments.

## Abstract

***Purpose:*** Poor survival rate of mesenchymal stem cells (MSCs) following their transplantation is one of the major challenges in their therapeutic application. Therefore, it is necessary to augment the viability of the MSCs in order to improve their therapeutic efficacy. Several strategies have been used to overcome this problem. Preconditioning of MSCs with oxidative stresses has gained a lot of attention. Therefore, in the present study, we investigated the effects of simultaneous preconditioning of MSCs with hydrogen peroxide and serum deprivation stresses on their survival and resistance to stressful conditions.

***Methods:*** MSCs were isolated from human umbilical cord blood. To perform simultaneous preconditioning, the cells were cultured in DMEM medium containing 1, 2.5 and 5 percent FBS and different concentrations of H_2_O_2_ (5, 10, 15, 20, 25, 30, 35, 40, 50, 60, 80 and 100 µM) for 24 hrs. Then, the cells were cultured in recovery culture medium. Finally, one group of the cells was exposed to a lethal concentration of H_2_O_2_ (300µM), and the other cells were cultivated in FBS free DMEM medium as the lethal situation. In addition, the percentage of apoptotic cells was analyzed using Caspase 3 assay kit.

***Results:*** Simultaneous preconditioning of the MSCs with 15µM H_2_O_2_ plus serum deprivation, 2.5% FBS, significantly increased the resistance of the cells to the toxicity induced following their cultivation in FBS free DMEM medium. It exerted the protective effect on the cells after treating with the lethal dose of H_2_O_2_ as well.

***Conclusion:*** Simultaneous preconditioning of MSCs with oxidative and serum deprivation stresses enhances their survival against harsh conditions, which might increase the viability and stability of the MSCs following their transplantation.

## Introduction

Recently, it has been clear that the mesenchymal stem cells (MSCs) are promising cell source for the treatment of a variety of human diseases^1,2^ including severe aplastic anemia^3,4^ acute graft- versus-host disease,^5^ cardiovascular diseases, acute liver failure^6^ and kidney injuries.^7,8^ Multi-lineage differentiation potential, immune modulatory properties and ability to localize specifically to injured sites have made MSCs as an appropriate alternative.^2,8^ According to animal and clinical studies, MSC transplantation can restore cardiac function probably by myogenesis and angiogenesis after myocardial infarction.^9-11^ Although there has been an increasing interest in using these cells in cell therapy but one of the main obstacles of their application is poor survival after transplantation. It has been shown that a majority of implanted cells die within few days after transplantation.^12,13^ Endogenous and environmental factors^14-18^ including inflammatory responses, lack of nutritional factors, hypoxia, reactive oxygen species (ROS) such as superoxide anion (O_2_-), hydroxyl radical (OH-), and hydrogen peroxide (H_2_O_2_), induce apoptosis and higher cell death either in vitro or in vivo MSCs microenvironments specially in ischemic heart medium.^16^ Hence, it is necessary to augment the viability of the MSCs in order to improve their efficacy. Moreover, several strategies including genetic manipulation and injection of growth factors and drugs have been employed to overcome this problem.^19^ In this regard, strategies which increase the survival of stem cells have gained significant attention. This indicates a need to understand underlying mechanisms of the decreased viability of the stem cells in the stress conditions.

Recently, preconditioning of MSCs with non-ischemic stresses such as stretch some chemicals, hypoxia, reactive oxygen radicals and oxidative stresses have been considered in the literature.^20,21^Previous studies have demonstrated that ischemic preconditioning (IPC) and oxidative preconditioning have protective effects on different kinds of cells and stem cells through pathologic condition and could be helpful to treat related diseases.^20-26^ Despite the importance of the effect of preconditioning, there have been no controlled studies on the harsh microenvironment of the injured tissues.

In order to potentiate MSCs against multiple threatening factors, it is necessary to expose them to several stresses conditions, which ensures their resistance against many inappropriate factors.^21,23,25,26^ Therefore, in the present study, the protective effects of co-preconditioning of MSCs with various concentrations of H_2_O_2_ and low doses of FBS have been evaluated. The main purpose of this study was to investigate the protective effects of simultaneous preconditioning on cell survival and prepare them to face with harsh microenvironments after transplantation.

## Materials and Methods

### Isolation and expansion of MSCs

The umbilical cord blood sample was collected from women who underwent caesarian section, with informed consent and mixed with citrate phosphate dextrose (CPD) anticoagulant. The sample was diluted in phosphate-buffered saline/ Ethylene diamine tetra acetic acid (PBS/EDTA) at a ratio of 3:1. Then, mononuclear cells were separated by Ficoll-Hypaque density gradient centrifugation (at 435g for 20 min), and seeded cell culture flasks containing low glucose Dulbecco’s modified eagle medium (DMEM), antibiotics (0.01% penicillin/ streptomycin) and 10% fetal bovine serum (FBS) (All of materials purchased from Gibco, Germany). The cells were incubated in the presence of 95% air and 5% CO_2_ at 37°C for 48hrs. Then, the non-adhered erythroid progenitor cells were removed by changing the medium. Medium refreshment was performed two times per week for 14 days prior to further studies. At 80% confluence, cells were detached with 0.25% trypsin-EDTA (Sigma Aldrich, Germany), washed with PBS and re-plated under the same culture conditions. To confirm the identity of the cultured umbilical cord blood MSCs (UCB-MSCs) morphologic features of the cells were evaluated by an inverted microscope and after that, the presence of specific surface markers, CD73, CD90 and CD105^27^ of MSCs were analyzed by flow cytometry device (Partc PASIII, Germany). The 4^th^ passages of UCB-MSCs were used in the present study.

### Preconditioning of MSCs

#### Preconditioning with H_2_O_2_

To investigate the possible cytoprotective effect of preconditioning with different concentrations of H_2_O_2_ on UCB-MSCs, 10000 cells were cultured in 96-well plate and then incubated with different concentrations of H_2_O_2_ (5, 10, 15, 20, 25, 30, 35, 40, 50, 60, 80 and 100 µM) for 24 hrs.^25^ followed by a recovery period of 12 hrs in usual growth medium. The experiment was examined in triplicate. After the recovery period, the culture medium was changed and preconditioned cells were exposed to 300 µM H_2_O_2 _as the lethal dose for 24 hrs.^25^

#### Preconditioning with serum deprivation (SD)

10000 UCB-MSCs were seeded in 96-well plates and preconditioned with 1, 2.5 and 5% FBS for 24 hrs followed by a recovery period of 12hrs in a usual growth medium. All of the concentrations were examined in triplicates. After the recovery period, the medium was decanted and the preconditioned cells were cultured in FBS free low glucose-DMEM for 24hrs as a lethal condition.

### Simultaneous preconditioning of MSCs with H_2_O_2_ and SD

In order to induce more stress condition to the cells and make them stronger against various toxic factors in the harsh microenvironment, the UCB-MSCs were cultured in oxidative stress and SD conditions. Briefly, the UCB-MSCs were cultured in 96-well plates containing low glucose-DMEM supplemented with 2.5% FBS, and simultaneously the cells were treated with 15 µM H_2_O_2_ solution for 24 hrs, followed by 12 hrs of recovery in a usual growth medium. After the recovery period, the cells separately were cultivated in serum-free condition or lethal dose of 300µM H_2_O_2 _for 24 hrs.

### Evaluation of cell viability

Cell survival of different preconditioned UCB-MSCs and control was assessed by colorimetric method using water-soluble tetrazolium salt-1 (WST-1) as described previously.^21^ Briefly, after treating of the cells with different stress conditions, the WST-1 reagent (Sigma, Germany) was added to culture media at a ratio of 1:10 and mixed gently. The plates were transferred to Co_2_ incubator at 37°C for 4 hrs. Using a microplate reader (BioTek, Germany), the optical density (OD) of each well was evaluated at 450 nm.

### Assessment of apoptosis

Caspase 3 activity was carried out using Caspase 3 assay kit (Sigma, Germany). According to manufacture protocol, cell lysate of different experimental and control groups was prepared. 5µl of the cell lysate and 5µl of Caspase 3 positive control were added to each well. Then, 10 µl of Caspase 3 substrate, and Caspase 3 inhibitor were added to the wells and incubated for 70-90 minute and finally**,** the absorbance was read at 405 nm with a microplate reader.

### Statistical analysis 

At each data point, the mean and standard deviation (SD) were calculated and statistically analyzed using Student’s t-test. *p*<*0.05 was considered significant.*

## Results

### UCB-MSCs were fibroblast- like and expressed general surface markers of MSCs

**Figure 1:**
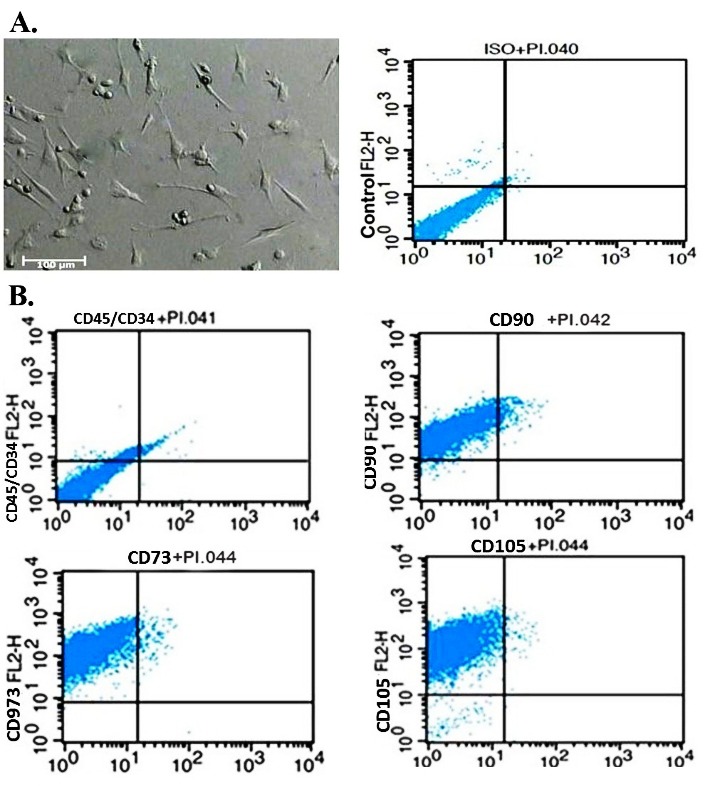
Characterization of umbilical cord blood mesenchymal stem cells (UCB-MSCs) A. Adherent UCB-MSCs displayed fibroblastoid morphology. B. Immunophenotype of isolated MSCs

Examination of the cells under an inverted microscope revealed that the isolated UCB-MSCs have fibroblast- like morphology and plastic adherent property (Figure 1A). Flowcytometry analysis was also employed to confirm the identity of UCB-MSCs. The results showed that the isolated cells were positive for CD29, CD105, and CD73 and negative for CD34 and CD45 (Figure 1B).

### H_2_O_2-_preconditioning enhanced cell survival of UCB-MSCs and decreased their apoptosis rate

**Figure 2:**
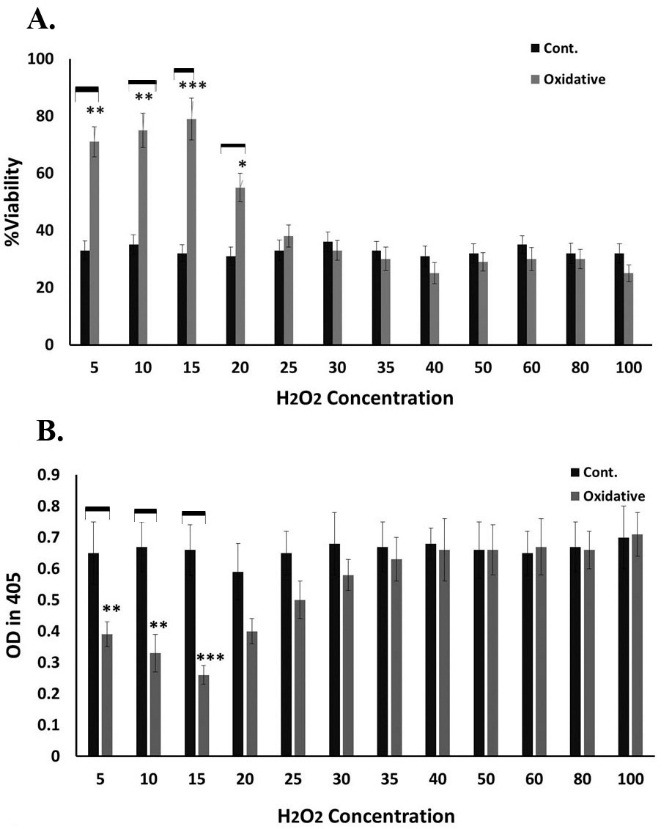
Evaluation of cell viability and apoptosis of H_2_O_2_-preconditioned UCB-MSCS following oxidative stress. A. WST-1 assay for analysis of cell viability. Higher survival was detected in the preconditioned cells in comparison with normal non-preconditioned cells (***p< 0.001, **p< 0.01, *p< 0.05). B. Assessment of Caspase 3 activity level as apoptosis indicator. H_2_O_2_-preconditioned UCB-MSCS exhibited lower Caspase 3 activity level in comparison with the related control groups (***p< 0.001, **p< 0.01). Data represents Mean ±SD of two independent experiments

As described in above, UCB-MSCs were treated by different concentration of H_2_O_2_. However, only preconditioning with 5, 10, 15 and 20 µM of H_2_O_2_ significantly increased the survival of UCB-MSCs in comparison with non-preconditioned control groups (without any treatment) following their exposure to lethal concentration (300µM) of H_2_O_2_ (p< 0.001 for 15 µM of H_2_O_2_ , p< 0.01 for 5 and 10 µM of H_2_O_2_ , and p< 0.05 for 20 µM). However, preconditioning with higher concentrations of H_2_O_2_ did not protect these cells from oxidative stress-induced cell death (Figure 2A). The results of the Caspase 3 level analysis were presented in Figure 2B. As is shown in Figure 2B, when 5, 10 and 15 µM H_2_O_2_-preconditioned-UCB-MSCs were exposed to lethal concentration of H_2_O_2_ (300µM) for 24 hrs obviously exhibited less Caspase 3 level, an index of apoptotic rate, in comparison with the non-preconditioned control cells, (p<0.001 for 15 µM of H_2_O_2_, p< 0.01 for 5 and 10 µM of H_2_O_2_). According to these data, 15µM H_2_O_2_ was considered as optimal dose for further studies.

### SD preconditioning protected UCB-MSCs from cell death and apoptosis in serum- free medium

**Figure 3:**
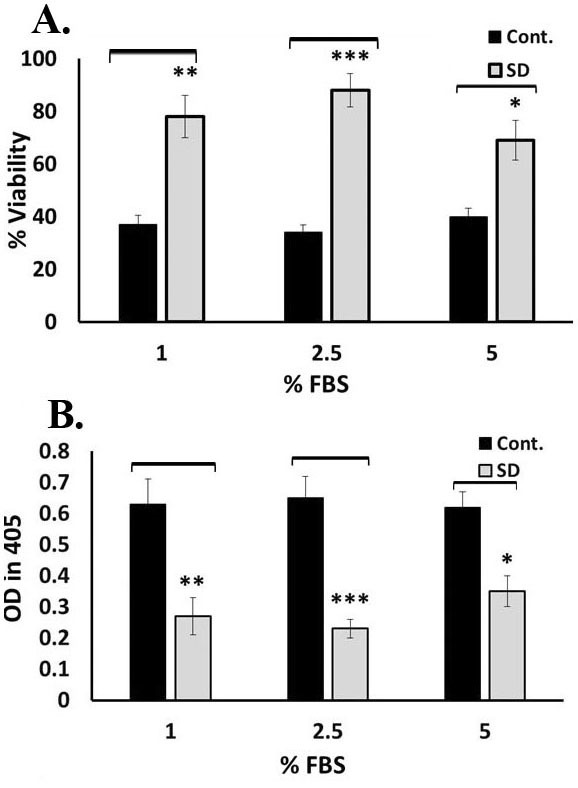
Assessment of cell viability and apoptosis rate of SD-preconditioned UCB-MSCS under serum free condition. A. WST-1 assay. (***p< 0.001, **p< 0.01, *p< 0.05). B. Assessment of Caspase 3 activity level. (***p< 0.001, **p< 0.01, *p< 0.05). subjected cells in compared to controls. Data represents Mean ±SD of two independent experiments

As is shown in Figure 3A and 3B, culturing of UCB-MSCs in medium containing 2.5 % FBS following cultivating under serum-free medium, as harsh stress- inducing condition, not only led to higher viability percentage in UCB-MSCs but also led to lower Caspase 3 activity level (p < 0.001) in comparison with the non-preconditioning group. P-value was 0.01 and 0.05 for the cells that were exposed to 1% and 5% FBS in comparison with control ( non-preconditioned cells) respectively. 2.5 % of FBS was set up as optimized serum deprivation condition and was considered as optimal dose for further studies.

### Simultaneous preconditioning of UCB-MSCs with H_2_O_2_ and serum deprivation conferred more resistance to these valuable cells against harsh condition

**Figure 4:**
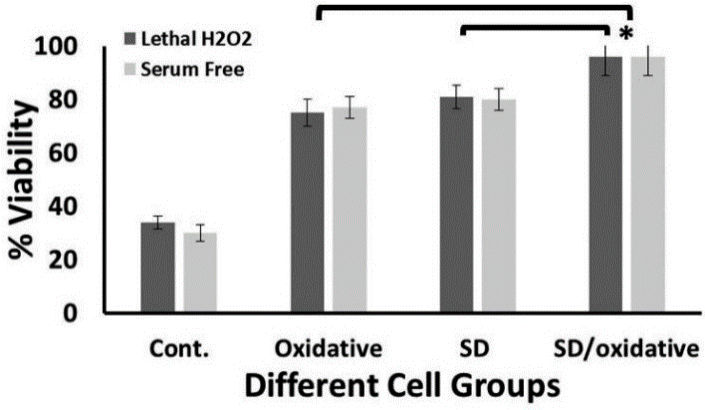
Viability of simultaneous (SD/Oxidative) - preconditioned UCB-MSCs after exposure to lethal oxidative stress and serum free condition. SD/Oxidative group was compared with those groups that were exposed to only SD or oxidative stress (*p< 0.05). Data represents Mean ±SD of two independent experiments

For induction of more stresses to UCB-MSCs, they were preconditioned with both 15µM H_2_O_2_ and 2.5% FBS. Then, they were cultured in 300µM lethal H_2_O_2_ or serum-free conditions. It is worthy to note that the viability of simultaneously preconditioned cells was higher than control and the groups receiving the single preconditioning modality (p<0.05). In other words according to the Figure 4, simultaneous preconditioning makes the UCB-MSCs** **more resistant to the harsh microenvironments.

## Discussion

Currently, the applications of the MSCs for cell therapy and tissue engineering purposes are under the focus of the investigation.^1,2^ However, many problems were developed in MSCs transplantation which hindered the prosperity of them in cell therapy^8,28^ Inappropriate environment of tissue containing high amounts of free oxygen radicals, inflammatory cytokines and lack of nutrition and blood supply are some factors which can threaten the survival of transplanted cells.^29^ Toma et al reported that less than only 1% of the cells survived few days after their transplantation into the heart of severe combined immune deficiency disorder mice.^12^ To solve this problem, several strategies like genetic modification of MSCs^19,30-32^ and injection of growth factors^33,34^ have been suggested in some literature. However, because of the risk of tumor development or low efficacy of these strategies,^19^ developing new strategies is necessary. The beneficial effects of preconditioning of the MSCs were first suggested by Murry et al, in 1986.^35^ Rosova et al reported that hypoxic preconditioning of MSCs led to the improvement of their healing potential.^36^ Also, Tang et al reported that preconditioning with H_2_O_2_ resulted in a reduction of apoptosis.^34^ In their study PC12 cell line, a rat cell line, was used. This group treated the cells with 0, 5, 10, 20, and 30 μmol L^−1^ of H_2_O_2_. After recovery by 24 hrs cultivation in normal media, these cells were exposed to 20, 30, 50, 100 μmol L^−1^ of H_2_O_2_ for another 24 hrs. They evaluated apoptosis rate by expression of Bcl2 level, mitochondrial membrane potential, and intracellular ROS^34^ Considering that the majority of engrafted MSCs may die within the first few days of transplantation, the transient cytoprotective effects of simultaneous preconditioning could be sufficient to protect transplanted cells during the first critical period after transplantation. Enhanced survival of implanted cells might reduce the required number of transplanted cells, which in turn, fewer stem cells may differentiate better.

Gargioli and colleagues exposed mouse perivascular myogenic progenitors to severe oxidative stress (200 and 400 μM of H_2_O_2_) and studied their survival, self-renewal and myogenic differentiation capacity. They reported that the H_2_O_2_-treated cells showed higher survival, proliferation and engrafted rate.^37^

The findings of the present study supported the hypothesis that preconditioning of MSCs might increase their survival and might increase their therapeutic potency for transplantation. Our results indicated that applying of an easy and non-expensive method can protect MSCs against the induced apoptosis by lethal stress conditions.

The molecular mechanism underlying the protective effects of different preconditioning methods on MSCs is not fully understood yet. However, lowering of apoptotic cells by regulation of some anti-apoptotic protein,^34^ modulation of some important growth factors/ cytokines/chemokines and their specific receptors and activation of signaling pathways such as Notch1/ Wnt11^38-40^ are the possible mechanisms.

Pretreatment with sub-lethal oxidative stress induces expression CXCR4 on the MSCs and enhances their survival as well as decreases apoptosis in these valuable cells.^25^ In the present investigation, we provided new evidence that simultaneous preconditioning of MSCs enhanced their survival rate. However, further and comprehensive studies should be performed to address the limitations of this study including the mechanisms underlying simultaneous preconditioning exert cytoprotective effects. Moreover, evaluation of apoptosis by other well-known techniques could be the subject of future studies. Finally, to expand the results of current study, *in vivo* study should be conducted to confirm the therapeutic potentialities in animal models.

## Conclusion

In conclusion, our study demonstrated that simultaneously preconditioning of MSCs with two different stresses enhanced protective effects. This would be a safe and versatile strategy to increase MCSs-based cell therapy in clinical applications.

## Acknowledgments

This study was supported by the Blood Transfusion Research Center, High Institute for Research and Education in Transfusion Medicine, Republic of Iran, grant number, 1413. The authors are thankful to Dr. Mahshid Mohammadipour for her technical assist.

## Ethical Issues

It was just *in vitro* study. The umbilical cord blood sample was collected with informed consent to separate MSCs.

## Conflict of Interest

The authors have no conflicts of interest.
